# Implications of Heterogeneous Biting Exposure and Animal Hosts on *Trypanosomiasis brucei gambiense* Transmission and Control

**DOI:** 10.1371/journal.pcbi.1004514

**Published:** 2015-10-01

**Authors:** Chris M. Stone, Nakul Chitnis

**Affiliations:** 1 Department of Epidemiology and Public Health, Swiss Tropical and Public Health Institute, Basel, Switzerland; 2 University of Basel, Basel, Switzerland; University of California, Los Angeles, UNITED STATES

## Abstract

The gambiense form of sleeping sickness is a neglected tropical disease, which is presumed to be anthroponotic. However, the parasite persists in human populations at levels of considerable rarity and as such the existence of animal reservoirs has been posited. Clarifying the impact of animal host reservoirs on the feasibility of interrupting sleeping sickness transmission through interventions is a matter of urgency. We developed a mathematical model allowing for heterogeneous exposure of humans to tsetse, with animal populations that differed in their ability to transmit infections, to investigate the effectiveness of two established techniques, screening and treatment of at-risk populations, and vector control. Importantly, under both assumptions, an integrated approach of human screening and vector control was supported in high transmission areas. However, increasing the intensity of vector control was more likely to eliminate transmission, while increasing the intensity of human screening reduced the time to elimination. Non-human animal hosts played important, but different roles in HAT transmission, depending on whether or not they contributed as reservoirs. If they did not serve as reservoirs, sensitivity analyses suggested their attractiveness may instead function as a sink for tsetse bites. These outcomes highlight the importance of understanding the ecological and environmental context of sleeping sickness in optimizing integrated interventions, particularly for moderate and low transmission intensity settings.

## Author Summary

Sleeping sickness, a disease that strikes predominantly poor populations in sub-Saharan Africa, has been targeted for elimination as a public health problem. Despite decades of control operations the disease remains enigmatic and is capable of persisting in populations at low levels of prevalence. Two mechanisms are investigated here that could allow persistence at such levels. Heterogeneous exposure of humans to tsetse is modelled as a subset of humans commuting to areas of high vectorial capacity. Additionally, non-human animals may act as reservoir species. We developed, parameterized, and investigated a model of sleeping sickness transmission to gain insight into the impact of these assumptions on the prospects of elimination using screening and treatment of humans and vector control. Supplemental use of vector control increased the probability of elimination and decreased the duration until elimination was achieved. This was more pronounced when animals did contribute to transmission, or when coverage and compliance of humans with screening operations was lower, for instance due to an inability to reach the humans at greatest risk of exposure. These results can provide insights to public health officials as to when to consider supplementing human treatment with additional measures, and thereby improve the prospects of elimination of this disease.

## Introduction

Human African trypanosomiasis (HAT), commonly called sleeping sickness, is a vector-borne and neglected tropical disease caused by two subspecies in the genus Trypanosoma, *Trypanosoma brucei gambiense* and *T*. *b*. *rhodesiense*. The main disease burden occurs in West and Central Africa due to *T*. *b*. *gambiense*. It causes a chronic disease which, with occassional exception, progresses over the course of months or years to a fatal meningo-encephalitis [1]. The gambiense form of HAT is typically thought of as an anthroponosis and transmission is predominantly by riverine tsetse, including species such as *Glossina palpalis* and *G*. *fuscipes*, which show a tendency to feed on human blood in locales where human-vector contact is high, such as water collection points [2].

HAT has been a disease of sporadically recurring epidemics with inter-epidemic periods of several decades, likely caused by historical contingency, poorly understood biotic factors, the breakdown of health services, or some combination of these [3,4], while at the same time being characterized by ancient, stable and geographically limited foci of transmission that may periodically lay dormant [5,6]. Recently, fewer than 10,000 cases per year are being reported [7]. On average, incidence and prevalence are therefore relatively low, but due to HAT’s highly focal nature, at a smaller scale, areas or villages may be heavily afflicted [2,8], and the existence of hidden pockets of high endemicity has been reported [9]. A recent study of the HAT situation in Uganda illustrated that while the disability-adjusted life years lost due to HAT at a national level was low compared to other infectious diseases, in highly affected districts the burden was comparable to the national average burden of diseases such as HIV and malaria [10].

Environmental or climatic changes appear to be shifting these foci in some areas [11], though the biotic and abiotic conditions that lead to an area being amenable to transmission are not well understood. Presence of vectors is necessary but not sufficient, because HAT is typically found only in restricted zones within the wider distribution of tsetse [3], suggesting that possibly high vector densities or particularly high human biting rates may be required. Aspects of human behavior may also contribute to transmission: for instance. Civil unrest (leading to a breakdown or inaccessibility of health care) and human cross-border movements are recognized as important factors leading to increases in, or maintenance of transmission [2,4].

To achieve elimination of HAT as a public health problem, it has been recognized that the available control measures of active case finding by mobile units (“screen and treat”), passive case detection through existing health facilities, and one or more methods of tsetse control, should be applied in a combination and at intensities most suited for particular epidemiological settings [12]. Mathematical models can prove particularly insightful for designing such potentially integrated control approaches [13,14,15,16], especially for rare diseases such as HAT where the interactions between multiple interventions can be difficult to tease apart in field trials.

Models of HAT transmission, typically based on the Ross-Macdonald style models of malaria [14,17,18], have struggled to capture the low prevalences associated with HAT without the addition of an animal reservoir or the immigration of infective tsetse from a connected area [17,19,20].

As a result of these modeling outcomes, and observations of the presence of *T*.*b*. *gambiense* infection in a range of vertebrates in certain foci [21,22], the existence of animal reservoirs has been posited. However, it is not clear whether the strains of *T*.*b*. *gambiense* circulating in non-human animals are the same as those found in humans—in one field study in Côte d’Ivoire humans and pigs were found to carry different genotypes of *T*.*b*. *gambiense* [23], and whether infection in animals contributes to transmission cycles or represents spillover from human communities. A recent modelling study supported the former view, but could not rule out that heterogeneity in exposure could likewise have led to similar infection patterns [20].

Bites by pathogen-carrying insects on humans are distributed in a non-uniform way [24]. The impact of a variety of forms of heterogeneity on vector-borne disease transmission has most notably been explored for malaria [25,26,27,28,29,30,31]. Factors responsible for heterogeneity in exposure that have been considered included differences in attractiveness of humans (as a result of sweat composition, body size and age, or even malaria infection status [32]), as well as spatial heterogeneity, for instance in the density of vectors due to distance from breeding sites, or behavioral patterns of humans that may bring them into closer or more prolonged contact with vector habitats. Heterogeneity of biting exposure and movement of humans, despite its putative involvement in HAT epidemiology, have rarely been incorporated in models. A notable exception was a two-patch model specifically investigating the role of villages and surrounding plantations and the consequences for an optimal allocation of vector control measures between these areas of activity [16].

There are indications that heterogeneous biting exposure may play a role in the transmission of human and animal trypanosomiases. For instance, herds of cattle living in the same village could have an exposure to tsetse (*G*. *m*. *submorsitans*) varying 5–10 fold from a homogenous estimate, due to the differences in tsetse densities within the spatial distribution of the cattle [33]. Specific areas within a focus of high transmission that present an even higher risk of exposure have been identified for HAT as well: for example, in the Bipindi focus in Cameroon, marshy hollows and areas near the river posed the greatest risk, in association with human behaviors such as washing or bathing, or agricultural activities [34]. In more urban areas of Kinshasa, where 66–67% bites of tsetse were reported to be on humans, likely due to the absence of alternative fauna, environmental risk factors such as raised areas with a mix of forest and river habitat, and the presence of pig sties were indicated for high transmission. A link to behavior that would bring people in contact with these habitats was implicated by the finding that 74% of cases reported were among farmers and fishermen [35].

Human movement patterns in concert with environment conditions are also thought to play a role: for instance, at a larger scale, the emergence of non-traditional (e.g., urban) foci may be linked to migrations associated with the expansion of coffee or cocoa plantations [35,36]. In a focus of Côte d’Ivoire, the confluence of human movement patterns, with a high degree of commuting from urban to rural parts but little mixing between rural areas, and environmental suitability for tsetse and the presence of certain ecotones favorable to human-tsetse contact in a specific part of the surrounding rural areas, resulted in a highly spatial heterogeneous transmission pattern [36].

Furthermore, there have been suggestions of clustering of infections within households. For instance, the strongest risk factor for *T*. *b*. *rhodesiense* infection in a case-control study in Uganda was a history of sleeping sickness in a member of the family, and proximity to wetlands was indicated as a significant spatial factor [37]. Similarly, evidence for familial clustering of *T*. *b*. *gambiense* was found in the Democratic Republic of Congo. Several hypotheses could support such clustering, for instance it could be related to the shared use of defined parts of a river, for washing or fishing, or interruption and resumption of an infective tsetse bite on a nearby human [38].

Investigations of heterogeneity have led to critical insights: the critical level of population coverage of an intervention required to bring R_c_ (the controlled reproduction number) below one, is lower when the interventions can be targeted, but greater when the high exposure groups cannot be identified or targeted [24]. Of potential relevance for HAT, Dietz [26] has shown that for a given value of *R*_0_, the prevalence of infection is lower when exposure is heterogeneous.

In this study, the goal was to account for the structural uncertainty regarding HAT epidemiology by developing a model that allows for both heterogeneous exposure of humans to tsetse and a possibility for animal hosts to contribute to transmission. The objective was to see whether and how these different assumptions affect the usefulness of two established control interventions against HAT: active case detection and vector control.

### Results

**Fig 1:**
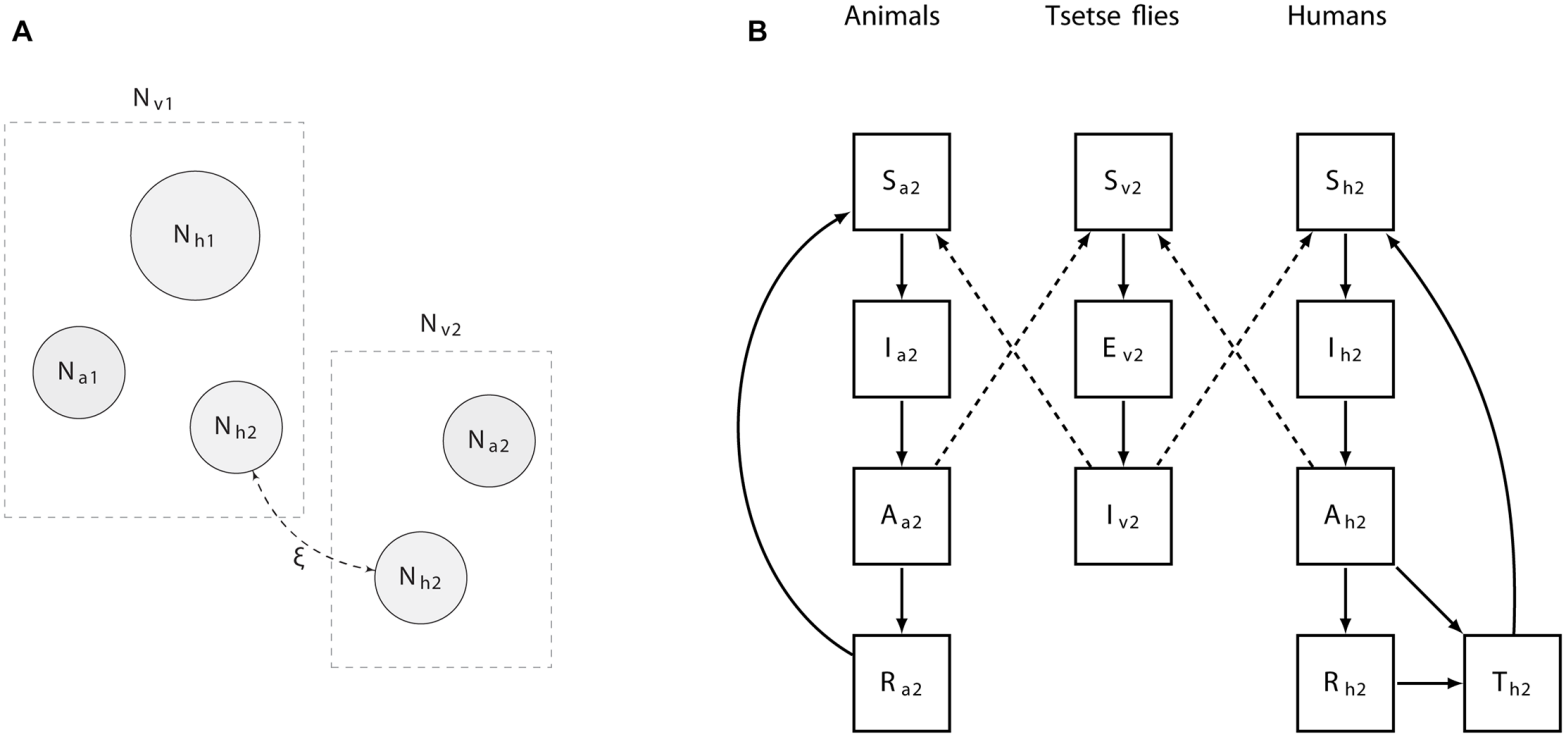
Overview of the population structure and compartments of the model. A): Human populations are divided in a stationary (N_h1_) population that remains in low exposure habitats (e.g., a village), and a smaller population (N_h2_) which commute and spend a proportion ξ of their time in a potentially high exposure setting (e.g., a plantation). Each of these habitats harbours tsetse (N_v1_ and N_v2_) and non-human vertebrate animal populations (N_a1_ and N_a2_) of varying sizes and characteristics. B): Compartmental diagram highlighting the transmissions between states of infection of the animal, tsetse, and human populations in the high exposure area 2. A similar diagram explains transmission in area 1, although there both human populations are exposed to tsetse bites. Solid lines depict transitions between compartments, while dashed lines represent transmission rates.

We developed a deterministic model of the West and Central African form (*T*. *b*. *gambiense*) of human African trypanosomiasis transmission. The model captures heterogeneity in exposure to tsetse bites, and can allow for the possibility of non-human animals to contribute to transmission. A schematic overview of the model structure is provided (Fig 1), while the details are given in the methods section, below. A description of all state and rate parameters is provided in Tables 1 and 2.

**Table 1:** Description of state parameters where *i* is 1 or 2.

*Parameter*	*Description*
S_vi_	susceptible vectors in area *i*
E_vi_	incubating vectors in area *i*
I_vi_	infective vectors in area *i*
N_vi_	total vector population in area *i* (sum of S_v_, I_v_ and E_v_)
S_hi_	susceptible humans of type *i*
I_hi_	incubating humans of type *i*
A_hi_	infective humans of type *i* (stage I of disease)
R_hi_	removed humans of type *i* (due to stage II of disease)
T_hi_	treated humans of type *i*
D_hi_	deaths in humans of type *i* due to sleeping sickness
N_hi_	total host population of type *i* available for bites (sum of S_hi_, I_hi_, A_hi_)
N^T^_hi_	total human population of type *i*
S_ai_	susceptible animals in area *i*
I_ai_	incubating animals in area *i*
A_ai_	asymptomatic animals in area *i*
R_ai_	recovered animals in area *i*

**Table 2:** Rate parameter descriptions, values used and ranges for model versions based on heterogeneity, but no animal reservoir.

*Parameter*	*Description*	*Unit*	*Prior range*	*Median values by transmission intensity*		
				High	Moderate	Low
μ_v_	Death rate of tsetse.	Day^-1^	[0.014–0.047]	0.030	0.037	0.024
f	Inverse of duration of feeding cycle.	Day^-1^	[0.2–0.5]	0.309	0.34	0.31
σ_h_	Biting preference for humans	-	[0–1]	0.326	0.40	0.33
σ_a1_	Biting preference for animal type 1	-	[0–1]	0.704	0.59	0.85
σ_a2_	Biting preference for animal type 2	-	[0–1]	0.396	0.63	0.009
ξ	Proportion of time spent in second region by commuters	-	[0–1]	0.698	0.64	0.61
b	Proportion of infective bites leading to infection in humans and animals	-	[0–1]	0.433	0.34	0.72
c_h_	Proportion of bites on an infective human that lead to a mature infection in flies	-	[0.0001–0.0051]	0.003	0.0031	0.0019
c_a1_	Proportion of bites on an infective animal of type 1 that lead to a mature infection in flies	-	[0.0001–0.0051]	0	0	0
c_a2_	Proportion of bites on an infective animal of type 2 that lead to a mature infection in flies	-	[0.0001–0.0051]	0	0	0
v_e_	1/extrinsic incubation period	Day^-1^	[0.025–0.0556]	0.042	0.037	0.037
η	Rate at which hosts move from the incubating stage	Day^-1^	[0.05–0.1]	0.07	0.085	0.099
s_1_	Rate of progression to stage II in humans	Day^-1^	[0.0012–0.0028]	0.002	0.0023	0.0016
s_ai_	Rate of progression to the immune class in animal hosts	Day^-1^	[0.0012–0.0028]	-	-	-
μ_s1_	Disease-induced death rate / rate of leaving the recovered state for humans	Day^-1^	[0.013–0.0029]	0.002	0.0021	0.0021
μ_h1_	Death rate of humans due to natural causes	Day^-1^	[3.4–6.8e-05]	4.3981e-05	6.3663e-05	3.7562e-05
μ_ai_	Death rate of animal host i	Day^-1^	[0.000164–0.0027]	0.0016/0.0019	0.0024/0.0011	0.0022/0.0025
r	Removal rate of infected humans due to treatment	Day^-1^	[0.0006–0.0044] ^†^	-	-	-
r3	Rate at which treated humans return to the susceptible class	Day^-1^	[0.014–0.14] ^†^	-	-	-
r4	Rate of loss of immunity in animal hosts	Day^-1^	[0.002–0.0054]	-	-	-
N_2_/N_1_	Ratio of humans in the high exposure environment to low exposure	-	[0–1]	0.032	0.0019	0.000194
V/H_i_	Number of vectors per human in area i	-	[0–10]	3.5/6.3	1.13/2.57	1.42/1.78
A/H_i_	Density of animals relative to humans in area i	-	[0–2]	1.35/0.54	1.31/0.91	1.25/1.81

^†^Values were zero (and not fit to prevalence levels) unless the interventions of screening and treatment of humans was simulated.

Since this system of equations can lead to a wide range of equilibrium prevalences when evaluated using a range of reasonable estimates for the rate parameters [39,40], we had to obtain parameter sets that allowed the behavior of the model around realistic prevalence levels to be examined. In active foci of HAT transmission, prevalences are often as low as 0.1–1% in humans [41]. Annual incidence thresholds associated with high (>1/10^3^ and <1/10^2^), moderate (> 1/10^4^ and <1/10^3^), and low (>1/10^5^ and <1/10^4^) risk categories of HAT have been suggested [42]. Based on the mid points of those ranges, thereby ignoring very high and very low outliers, an assumption that incidence will be comparable to prevalence if mobile units visit afflicted areas infrequently, and an underreporting rate of approximately 3 [12], we specified prevalence levels of 1.65%, 0.165%, and 0.0165% as being representative of high, moderate, and low transmission settings, respectively. A critical assumption we made is that these prevalence levels were stable. We note that due to the paucity of available data, posterior parameter estimates were unlikely to change significantly from the prior ranges. The intent, however, was not to obtain more precise parameter estimates, but rather to act as a filter for sets of parameters that would lead to unrealistic prevalences. We obtained parameter sets using a Bayesian framework of importance resampling [43,44].

Median values of the resampled estimates for the animal reservoir and no animal reservoir versions at high, medium and low transmission intensities are provided (Tables 2 and S1, respectively). For both the version with and without the possibility of an animal reservoir, as transmission intensity decreased (i.e., from the high to moderate to low intensities), the number of parameter sets in the resampled sets did as well, presumably because the parameter space leading to such very low prevalence levels was sparser than that leading to high levels of prevalence. Stable levels of sleeping sickness prevalence associated with high, moderate, and low transmission in field settings were found using both the model version without the possibility of transmission from non-human animals to tsetse, and the model version allowing for animal reservoirs. In both cases a role for heterogeneity in exposure to tsetse bites was suggested, whereby the smaller the proportion of humans at high risk, the lower prevalence could be, particularly in the absence of an animal reservoir. For instance, the estimated median ratio of humans commuting to an area with potentially higher tsetse biting rates (N_2_/N_1_) was 0.03 (without animal reservoirs) and 0.06 (with animal reservoirs) in the high transmission settings.

To gain insight into the parameters that drive HAT prevalence at the levels encountered in the field, we performed a global sensitivity analysis. We created 500 parameter sets by drawing Latin hypercube samples for each parameter from uniform ranges between the largest and smallest of the median values estimated for the high, moderate and low transmission settings for the model versions with (S1 Table) and without animal-tsetse transmission (Table 2). We simulated the equations until the asymptotically stable equilibrium point was reached and the prevalence of infection in humans was recorded for each iteration. The sensitivity of prevalence to model parameters was investigated for 500 samples by calculating the partial rank correlation coefficients, which gives an indication of the degree to which an output parameter is related to an input parameter when controlling for the effects of other parameters. The sensitivity analyses were performed using SaSAT [45].

**Fig 2:**
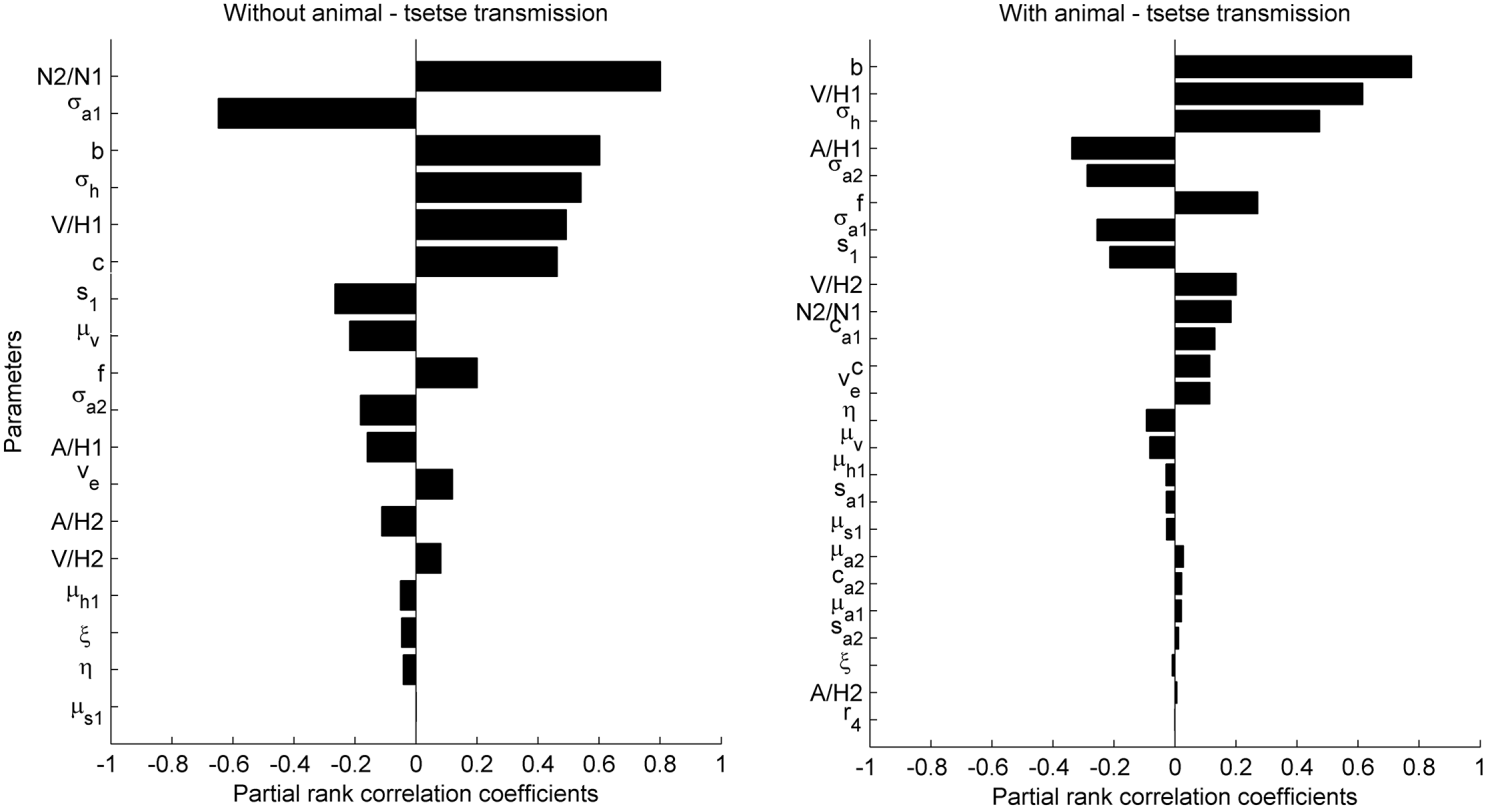
Global sensitivity of sleeping sickness prevalence to model parameters without (left panel) and with the possibility of animal-tsetse transmission of *T*.*b*. *gambiense* (right panel). Descriptions of parameters are provided in Table 2. The most important parameters without an animal reservoir were the ratio of commuters to non-commuters (N_2_/N_1_), the biting preference of tsetse for animals in the non-commuting area, *σ*_*a1*_, and the proportion of infectious bites that lead to infection in hosts, *b*. With an animal reservoir, the most important parameters were b, the tsetse to human ratio in the focal (1^st^) area, V/H_1_, and the biting preference for humans, *σ*_*h*_.

The sensitivity of sleeping sickness prevalence to model parameters sheds light on the drivers of heterogeneous exposure (Fig 2). In the case where non-human animals cannot contribute to transmission directly, the parameters with the greatest influence on prevalence were the proportion of humans commuting to this area (N_2_/N_1_), and the tsetse biting preference for peridomestic animal hosts (*σ*_*a1*_). The density of vectors to humans in the village (V/H_1_), the biting preference for humans (*σ*_*h*_), as well as parameters related to transmission efficiency (*b*, *c*) were also of importance.

When allowing for animal-tsetse transmission a number of the same components stand out as drivers of prevalence, namely the efficiency of transmission to humans and animals (*b*), the vector density in the village (V/H1), and the biting preference for humans (*σ*_*h*_).

**Fig 3:**
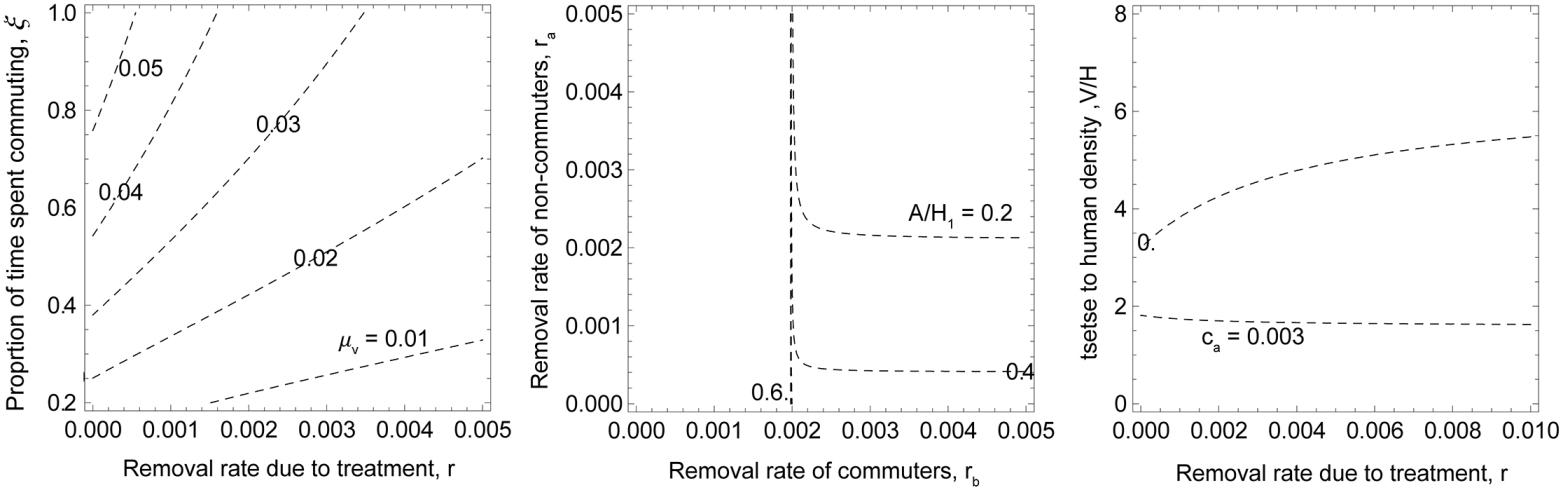
Zero-growth isoclines (*R*_0_ = 1) of *T*.*b*. *gambiense* under perturbation of specific parameters. The parameter values used were the median values obtained for the high transmission setting, except for those varied in the analysis. In the left plot, isoclines at different levels of vector mortality are shown, depending on the daily removal rate of infected humans (*r*) and the proportion of time commuters spend in the high exposure area (ξ). The areas above the isoclines represent values of *R*_0_ greater than 1, and below and to the right of the isoclines values smaller than 1. In the middle plot the impact of screening humans in the low risk setting (*r*_*a*_) in combination with screening commuting humans (*r*_*b*_) is shown for different levels of animal to human ratios (A/H_1_). In the right plot isoclines are depicted along removal rates (*r*) and tsetse density (V/H) in both areas when animals either do not contribute to transmission (*c*_*a*_ = 0) or they can infect tsetse (*c*_*a*_ = 0.003).

To gain further insight into how the efficacy of different parameters and potential control approaches (screen & treat and vector control) depend on variation in other parameters, we investigate the impact on the basic reproduction number, *R*_0_, by looking at zero-growth isoclines, i.e., the parameter space where *R*_0_ = 1 (Fig 3). As the human populations are more separated (with increasing ξ) a greater removal rate is required to interrupt transmission, and removal rates leading to *R*_0_ < 1 become smaller as vector mortality increases (left plot). When we compare the impact of screening humans in the low risk setting (*r*_*a*_) and screening commuting humans (*r*_*b*_), it is clear that screening the humans commuting to high exposure areas is critical, as transmission can be sustained by this group even at very high rates of screening the non-commuting population (*r*_*a*_). Depending on the density of animals in the low risk area (which here do not contribute to transmission, but can lead to “wasted bites” among tsetse), it can be sufficient to target only the commuting population. When animal densities are low, resulting in higher biting rates on humans in the village (N_h1_), then transmission could also be sustained among this population and all populations will need to be screened (middle plot). When animals can contribute to transmission (*c*_*a*_ = 0.003), treatment of infected humans will not lead to interruption of transmission above a certain vector-human density threshold, and tsetse control will have to be relied on (right plot).

In addition to the feasibility of interrupting transmission at various levels of efficacy, the timelines under which prevalence decreases to near zero can also be a relevant consideration. Simulations were performed to assess these timelines and probabilities, allowing for the range of model parameter uncertainty, as well as a range of uncertainty in control intervention efficacy, and structural model uncertainty regarding the ability of animals to contribute to sleeping sickness transmission.

Note that when elimination is used in this context, it is strictly more accurate to speak of elimination as a public health problem. This is because we used a deterministic model and had to specify an arbitrary threshold below which we assume elimination is likely in reality to occur. We used a prevalence in humans of < 1 x 10^−6^, below the lowest threshold associated with very low risk HAT foci [42]. A stochastic model allowing for immigration and importation of infections, with discrete numbers of infected individuals, would be required to investigate actual elimination. For these simulations we investigate high transmission settings only, as these are the ones where *a priori* a combination of interventions may be considered [12]. A wider range of interventions and their cost-effectiveness using this modelling approach over all transmission settings will be reported on elsewhere (Sutherland et al, in prep).

The interventions we considered were case detection in humans (screen and treat), potentially supplemented with vector control using targets or traps. The effectiveness of traps may differ by the species of tsetse and environmental conditions, as well as issues related to spacing and maintenance. Estimates from the literature suggest attaining a 5% daily mortality is often attainable [46,47,48], and we used a broad range of 1–10% to capture this variability. We assume screen and treat operates by removing 1^st^ and 2^nd^ stage infected people at a daily rate. We use the formula specified by Artzrouni & Gouteux [14] who relate a percentage, *d*, of humans effectively screened in a given period (a month in their model, here, a year):

$$d\;=\;100\left(1-e^{-365\;r}\right)$$

and the daily removal rate, *r*, (which appears in eqs (3) through (5) and (8) through (10)) is therefore:

$$r\;=\;-\text{ln}\left(1-\frac{d}{100}\right)/365.$$

The percentage of humans screened at a yearly basis will depend on the frequency of visits of mobile teams, as well as other factors such as the achieved level of coverage and compliance, and sensitivity of the diagnostic test. Here, we investigate a broad range of 20 to 80% of the population. In one analysis where we investigate the impact of having different screening rates on humans in low and high exposure populations, instead of a single parameter *r*, we then specify *r*_*a*_ and *r*_*b*_ for the removal rate of N_h1_ and N_h2_, respectively.

**Fig 4:**
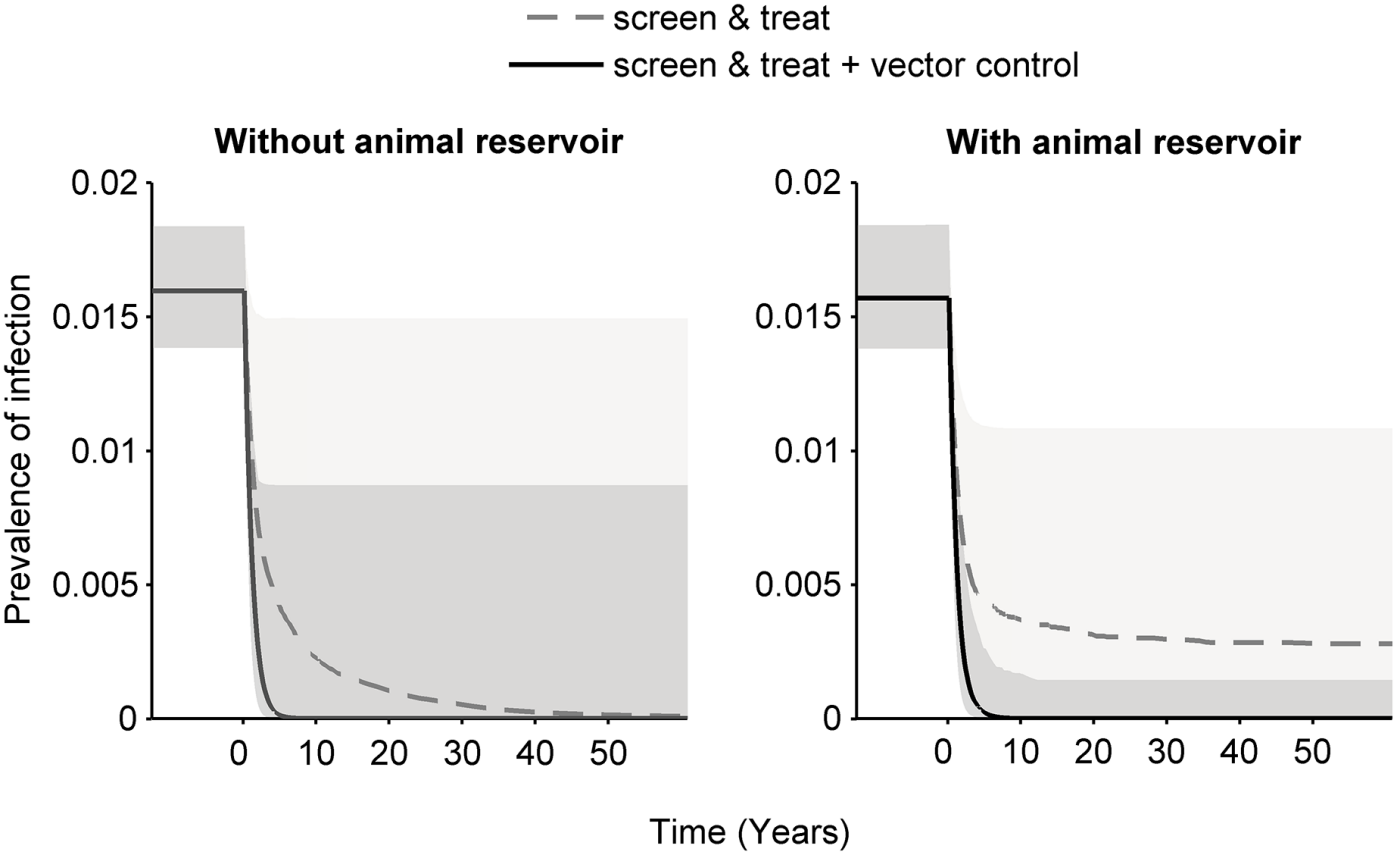
Median (lines) and 95^th^ percentiles (shaded areas) of simulations on the impact of interventions on prevalence over time in high transmission settings without (left) and with (right) animal-tsetse transmission, assuming a range of efficacies for screen & treat (solid line) and screen & treat with vector control (dashed line).

On average, these simulations suggest that if animals do not contribute to transmission, deployment of either a screen & treat strategy or an integrated approach of screening together with vector control is likely to result in interruption of transmission (Fig 4). However, the rate at which prevalence declines toward zero is considerably faster when using the integrated approach. If biting an animal can result in a tsetse becoming infected, screening and treatment of humans by itself is less likely to lead to interruption of transmission, although prevalence in humans will still be reduced.

**Fig 5:**
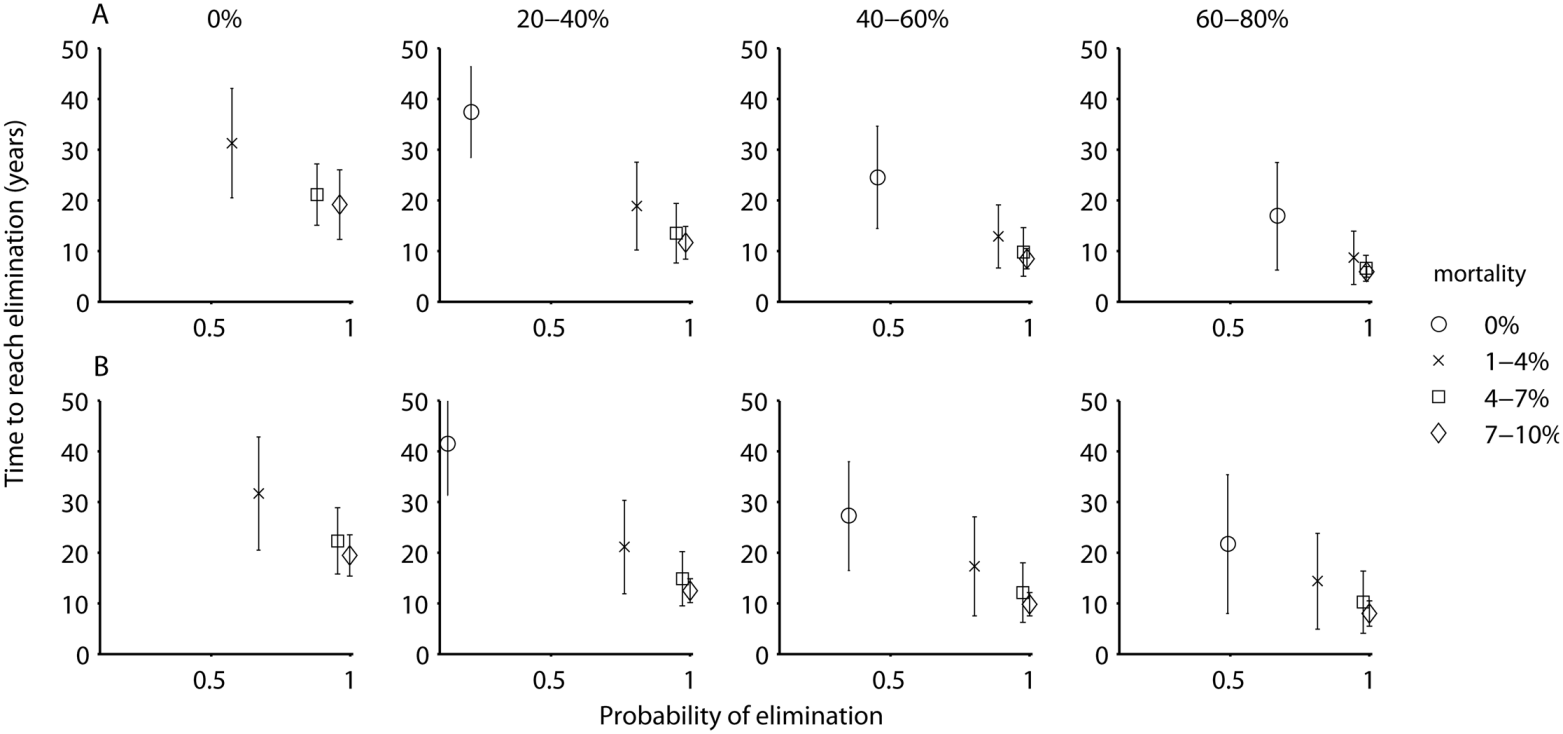
The proportion of simulations where HAT was eliminated (prevalence < 1 x 10^−6^) and the mean time to elimination with standard deviation, depending on the percentage of the human population screened per year (expressed as absent (0%), low (20–40%), moderate (40–60%), or high (60–80%) coverage and indicated by the plot titles), with varying levels of vector control (expressed as additional vector mortality, indicated by the symbols in the legend), for a high transmission setting without an animal reservoir (A) and with an animal reservoir (B).

To explore the potential of using an integrated approach of screening and treatment with vector control in more detail in different transmission settings, and depending on whether animals were assumed to to be capable of transmitting infections, we ran sets of 500 simulations whereby both approaches were varied from absent, to low, moderate, or high coverage. For each set we determined the mean time required to reach elimination (i.e., a prevalence < 1 x 10^−6^) and the proportion of 500 simulations that led to elimination. In the high transmission setting in the absence of animal reservoirs, to achieve the goal of elimination within 20 years and with greater than 80% probability, either vector control at the highest level of efficacy (resulting in a mean time to reach elimination of 19 years), or an integrated approach of screen & treat and vector control was required. A combination of both at our lowest levels of efficacy (20–40% coverage for screening, and a mortality rate between 1–4% for vectors) was sufficient to reach this threshold, and either of the two interventions could be intensified in order to achieve greater probabilities of success or to reduce time to elimination. In general, increasing the intensity of vector control led to greater increases in the probability of eliminating, while increasing the intensity of human screening led to a sharper reduction in the time required to eliminate (Fig 5).

**Fig 6:**
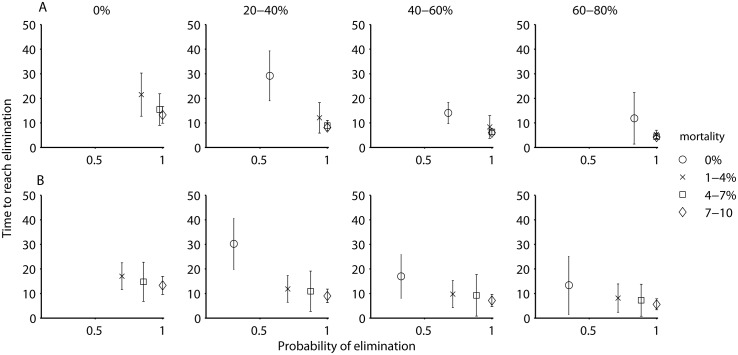
The proportion of simulations where HAT was eliminated (i.e., prevalence < 1 x 10^−6^) and the mean time to elimination, depending on the percentage of the human population screened per year, with varying levels of vector control (expressed as additional vector mortality, indicated by the symbols in the legend), for a moderate transmission setting without an animal reservoir (A) and with an animal reservoir (B).

**Fig 7:**
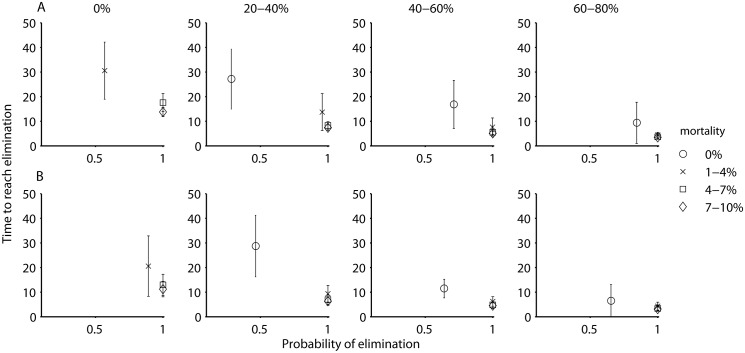
The proportion of simulations where HAT was eliminated (i.e., prevalence < 1 x 10^−6^) and the mean time to elimination, depending on the percentage of the human population screened per year, with varying levels of vector control (expressed as additional vector mortality, indicated by the symbols in the legend), for a low transmission setting without an animal reservoir (A) and with an animal reservoir (B).

In both the moderate and low transmission settings the time required to reach elimination was on average shorter, and the patterns overall were similar. In these settings, while an integrated approach resulted in high probabilities of achieving elimination within the shortest period, both approaches (screen and treat and vector control) employed by themselves at the high levels of efficacy also resulted in elimination, albeit at a somewhat lower probability and over a longer period (Figs 6A and 7A).

If there are foci where animal reservoirs do contribute to transmission, the patterns are in general similar to those from the model that assumes no animal reservoir, except that the probability of eliminating without vector control is reduced (Figs 5B, 6B and 7B). The conclusions are similar in regard to an integrated approach of screen & treat and vector control likely being necessary to eliminate with a greater than 80% probability and within 20 years, although a strategy of vector control by itself may also be acceptable, especially in moderate or low transmission zones.

## Discussion

Increased understanding of the ecological, environmental and behavioral drivers of HAT transmission is critical to designing effective control programs that maximize the probability of achieving elimination. In this study we focused on the role of non-human animals and the importance of heterogeneous exposure of humans to infected tsetse bites. A key insight is that regardless of whether or not non-human animals contribute to the transmission cycle of *T*. *b*. *gambiense*, animal populations play a crucial, but varying, role in the epidemiology of HAT.

Although the consensus currently suggests that non-human animals do not play an important role in HAT transmission, we found that when—in perhaps a minority of foci—animal populations are capable of harboring *T*.*b*. *gambiense* and infecting tsetse, then a strategy based only on case detection and treatment of humans is less likely to lead to interruption of transmission, and additional vector control interventions are likely necessary. We did not disentangle the factors that allowed screen & treat to interrupt transmission when animal-tsetse transmission could occur, but speculate that whether animal populations constitute a reservoir will depend on a combination of their efficiency of transmission, densities of animals and tsetse flies, and contact patterns of humans. It is perhaps worth noting that the current thought in HAT epidemiology that animals do not contribute to transmission is based largely on experiences where screening and treatment of human populations alone, without vector control, has led to the local elimination of HAT in certain foci (e.g., [49]). However, it may be dangerous to base such conclusions on limited natural experiments. Our simulations suggest that even if animals can contribute to transmission, whether vector control is required may depend on more specific epidemiological or ecological factors. For instance with animal-tsetse transmission, at the highest rate of screening without vector control, the probability of achieving elimination varied between 0.35–0.65 for the different transmission settings (Figs 5A, 6A and 7A). Further validation of these results is required, but what likely occurred in our model was that although animals could contribute to transmission in these simulations, their type reproduction number in a subset of the parameter sets may have been below 1, and maintenance may have been due to humans. In the other subset of parameter values, it appears that infection in humans may have been due to spillover from animals.

When we did not allow for animal-tsetse transmission in our model—a common assumption in models of *T*. *b*. *gambiense* transmission [18,40]—the role of animal populations was one of dilution or zooprophylaxis: essentially leading to tsetse “wasting” bites on inefficient hosts [50]. Similar insights were derived in a previous modelling study of *T*. *b*. *rhodesiense*, where in certain areas between 50–90% of meals may be taken by *G*. *fuscipes fuscipes* on monitor lizards, which are incompetent hosts [15]. This was evident from a comparison of the sensitivity analyses for both model versions. Without animal-tsetse transmission, one of the most important parameters with a negative relation to prevalence was a preference for tsetse to bite animals instead of humans in the area surrounding the main human population. In a similar study, although with different equations for the host choice model, Davis et al [40] found comparable outcomes where the most important parameters in a sensitivity analysis of *R*_0_ were the proportion of bites on humans, followed by the susceptibility of *Glossina fuscipes* to infection, the tsetse to human ratio, and vector mortality.

Under this scenario where only humans can transmit infection to tsetse, our results suggest that an integrated approach of screening humans along with vector control will be the most efficacious and perhaps required: none of the screening coverage levels without vector control resulted in > 80% of simulations leading to elimination in the high transmission setting, whereas adding even the lowest level of vector control led to large increases in the probability of eliminating and reduced the time required to do so. Additionally, increasing the intensity of vector control was more likely to eliminate HAT transmission, while increasing the intensity of human screening reduced the time to elimination. Whether adding vector control is cost-effective however will require model extensions that include health impacts and implementation costs, and will be explored in a follow-up study on control and elimination strategies (Sutherland et al, in prep).

We found that if a strategy for interrupting transmission only employs human case detection, it is necessary and sometimes sufficient that those humans who are at the highest risk, i.e., exposed to higher levels of tsetse bites, can be reached effectively. The opposite, an inability to target high exposure groups, which would hamper the ability to interrupt transmission [24] which may occur, for example, if people working in areas of high tsetse densities, like plantations, are less likely to attend mobile screening events due to opportunity costs [51,52]. Our outcomes thus suggest that in order to better identify risk factors leading to heterogeneity in HAT transmission and determine their implications for control strategies, understanding the regulation of tsetse population dynamics and biting behavior in relation to humans and non-human animals, as well as the suite of human activities that lead people to visit high risk areas, should be a priority. Similarly, measuring the efficiency with which animals can infect tsetse under natural field conditions would greatly reduce the structural uncertainty in our understanding of HAT transmission. The latter may also have important implications for optimizing elimination strategies by transmission setting: at the high levels, regardless of the presence of an animal reservoir, our results support an integrated approach. At the moderate and low levels, the conclusions are less straightforward: if an animal reservoir does not exist, either human treatment or vector control by themselves may be sufficient and still allow for elimination to be reached with reasonable probabilities and within a reasonable timeframe, whereas if an animal reservoir does exist, vector control by itself may be sufficient, but relying on human treatment alone likely will not be.

There are several important assumptions implicit in this modelling study that could affect our outcomes. The median level of heterogeneity in our parameterizations is high compared to the common assumption that 20% of the people account for 80% of transmission [24]. However, prevalence data is often recorded at larger spatial scales (such as provinces or districts) than the scales at which transmission occurs (such as villages and hamlets). One such example of heterogeneity comes from a description of epidemiological data in the Bipindi focus (of high transmission), where 95% of cases were located in only 2 out of 15 villages [20] within the focus. In addition to this spatial heterogeneity, the behavioral and environmental factors described in the introduction would further increase the heterogeneity in exposure. It is possible that other factors, for instance the occurrence of long-term asymptomatic or chronic carriers [1,53], may also have important effects on HAT transmission. Recently evidence for very long term asymptomatic carriage in stage I has been reported [54]. However, it remains unclear how common such occurrences are. In our model we assume a constant rate of progression from stage I to stage II. This leads to an exponential distribution of time spent within the stage, with a tail of a few people who would be expected to remain infectious for a very long time [53]. In the absence of better data on the frequency with which these chronic carriers occur, we thus assume that the extent to which they occur in our model based on an exponential distribution is reasonable. Additionally, the impact of heterogeneity, as well as the animal host species and other factors may vary between foci. Model fitting to data (as it becomes available) that encompasses human movement and activities as well as heterogeneity in tsetse density and biting behavior would be required to reduce parameter uncertainty beyond that which we were able to do here, and corroborate the importance and extent of heterogeneity in HAT transmission.

We also assumed that prevalence of HAT was at a stable equilibrium in the absence of control interventions, which is at odds with historical data on a number of epidemic resurgences of the disease. The reasons for which are unclear, although they have been linked to ecological and societal disruptions caused by colonization [55]. Furthermore, our current model for screen & treat and vector control may ignore essential spikes and lapses in coverage by translating into continuous rates, what are in reality pulses associated with the roll-out of mobile teams or the set-up of tsetse traps. We have also made the assumption that passive detection of cases through the regular health only occurs after they have progressed beyond stage I, i.e., that passive detection has no effect on transmission. If this view proves to be too pessimistic, a baseline value for the removal rate of humans due to case detection, *r*, greater than zero should be used. As longitudinal data of both prevalence in humans, animals, tsetse, and the performance of both the active and passive systems in specific foci becomes available, model fitting should take these considerations into account.

Additionally, we simulated vector control by increasing the daily mortality rate of tsetse, but not decreasing their abundance, likely underestimating the impact of tsetse control. A more detailed model could include tsetse population dynamics [56] and the impact of seasonality (in tsetse population dynamics, human behavior, and control interventions) and could be extended to include temperature-dependent tsetse life history traits [57].

Although we included a metapopulation structure at a small scale (e.g., village and plantation), we did not allow for connectivity between different foci and resulting migration of either infected humans, animals, or tsetse [58]. Considering a larger spatial scale and movement patterns would allow for reinvasion following local interruption of transmission and likely make elimination more difficult to achieve than the results presented here. We assumed that areas at all ranges of transmission intensities allow for transmission in the absence of immigration of infected hosts, but if in reality there are instead sources (active foci with high transmission) and sinks (areas with (very) low intensity that cannot sustain transmission), then this would have obvious implications for elimination strategies. A large scale metapopulation modeling study would be a useful follow-up to our current work. It should be noted however that including such additional sources of complexity will come at a cost of model tractability, i.e., understanding the model drivers becomes more difficult and idiosyncratic results harder to explain. Likewise, data fitting and validation against different data sets may become more difficult; and before adding more complexity, it may be prudent to perform such validation exercises with the current model. One area where additional fitting and validation may shed light is the very low to low transmission intensity settings. As indicated, in the version without animal reservoirs, our fitting procedure led to repeated sampling of very few parameter sets for this transmission setting. This may be an indication of a model struggling to capture these patterns, and could indicate the existence of a reservoir of some type (whether this is an animal reservoir, or migration of infected cases from nearby higher prevalence zones). Until further data fitting and validation can be undertaken, the results for the low prevalence setting should thus be interpreted with care.

In conclusion, these results show the potential importance of heterogeneous exposure of humans to tsetse bites, and interactions between abundance and attractiveness of non-human animals to tsetse, as important drivers of HAT epidemiology, even when animals do not constitute hosts for *T*. *b*. *gambiense*. An increased understanding of these ecological dimensions of sleeping sickness is not only expected to lead to a better understanding of risk factors for transmission, but also to tailoring foci-specific integrated and cost-effective control approaches.

## Methods

### Model

Our deterministic model of human African trypanosomiasis (*T*. *b*. *gambiense*) transmission is defined by a system of ordinary differential equations for compartments of two tsetse, animal and human populations, indicated by subscript *i*, where *i* below is 1 or 2. The state variables and rate parameters of the model are described in Tables 1 and 2, respectively. We denote each compartment with upright upper case Latin letters and the total number of individuals in each compartment by italicized upper case Latin letters.

For humans, we have susceptible (S_hi_), incubating (I_hi_), and two compartments related to the distinct stages of sleeping sickness, the 1^st^ or asymptomatic stage (A_hi_), and the 2^nd^ stage where trypanosomes have reached the cerebro-spinal fluid (R_hi_). Finally, we consider a treated compartment (T_hi_), predominantly to enable downstream application of the model to health impact and cost-effectiveness studies. The total human population is given by:

$$N^{T}{}_{hi}\left(t\right)\;=S_{hi}\left(t\right)\;+I_{hi}\left(t\right)\;+A_{hi}\left(t\right)\;+R_{hi}\left(t\right)\;+T_{hi}\left(t\right).$$

We incorporate the assumption made by Artzrouni & Gouteux [18] that humans in advanced stages of the disease (R_hi_) or those receiving treatment (T_hi_) are not available to tsetse bites, and therefore the population sizes used to calculate biting preferences exclude these two compartments,

$$N_{hi}\left(t\right)\;=S_{hi}\left(t\right)\;+I_{hi}\left(t\right)\;+A_{hi}\left(t\right).$$

To better understand the role of animals as reservoir hosts, we develop two separate models. In the first model, we assume that trypanosomes cannot infect animals and model two animal populations as constant parameters, N_ai_. In the second model, we divide the animal populations into susceptible (S_ai_), incubating (I_ai_), infectious (A_ai_), and recovered (R_ai_) classes. We adopt the assumption that animals can recover from infection and are then temporarily immune, R_ai_, before returning to the susceptible state [17]. The total animal populations are,

$$N_{ai}\left(t\right)\;=S_{ai}\left(t\right)\;+I_{ai}\left(t\right)\;+A_{ai}\left(t\right)\;+R_{ai}\left(t\right).$$

In both models, we assume that all animals are subject to bites by tsetse files. For tsetse, we have susceptible (S_vi_), incubating or latent (E_vi_) and infective (I_vi_) compartments, so that the total vector population is given by:

$$N_{vi}\left(t\right)\;=S_{vi}\left(t\right)\;+E_{vi}\left(t\right)\;+I_{vi}\left(t\right).$$

The equations describing the changes of numbers in human compartments (Fig 1) are given by:

$$\frac{dS_{h1}}{dt}=\beta_{h1}+\;r_{3}T_{h1}-\;\mu_{h}S_{h1}-\frac{b\;f\;\theta_{h1,1}\;I_{v1}\left(t\right)}{N_{h1}}\;S_{h1}\left(t\right),$$ (1)

$$\frac{dI_{h1}}{dt}\;=\frac{b\;f\;\theta_{h1,1}\;I_{v1}\left(t\right)}{N_{h1}}\;S_{h1}\left(t\right)\;-\;(\mu_{h}+\eta )\;I_{h1},$$ (2)

$$\frac{dA_{h1}}{dt}=\;\eta_{}\;I_{h1}-\;\left(\mu_{h}+s_{1}+r\right)A_{h1},$$ (3)

$$\frac{dR_{h1}}{dt}\;=\;s_{1}A_{h1}-\;\left(\mu_{h}+\mu_{s1}+r\right)R_{h1},$$ (4)

$$\frac{dT_{h1}}{dt}\;=\;rA_{h1}+\;rR_{h1}-\;\left(\mu_{h}+\;\mu_{t}+r_{3}\right)\;T_{h1},$$ (5)

$$\frac{dS_{h2}}{dt}\;=\;\beta_{h2}+\;r_{3}T_{h2}-\;\mu_{h}S_{h1}-b\;f\;(\frac{\;\theta_{h2,1}I_{v1}\left(t\right)}{N_{h2}}+\;\frac{\theta_{h,2}\;I_{v2}\left(t\right)}{N_{h2}})\;\;S_{h2}\left(t\right),$$ (6)

$$\frac{dI_{h2}}{dt}{\;}_{\;}=\;b\;f\;(\frac{\;\theta_{h2,1}I_{v1}\left(t\right)}{N_{h2}}+\;\frac{\theta_{h,2}\;I_{v2}\left(t\right)}{N_{h2}})S_{h2}\left(t\right)\;-\;(\mu_{h}+\eta )\;I_{h2},$$ (7)

$$\frac{dA_{h2}}{dt}=\;\eta \;I_{h2}-\;\left(\mu_{h}+s_{1}+r\right)A_{h2},$$ (8)

$$\frac{dR_{h2}}{dt}\;=\;s_{1}A_{h2}-\;\left(\mu_{h}+\mu_{s1}+r\right)R_{h2},$$ (9)

$$\frac{dT_{h2}}{dt}\;=\;rA_{h2}+\;rR_{h2}-\;\left(\mu_{h}+\;\mu_{t}+r_{3}\right)\;T_{h2}.$$ (10)

The population size is assumed to be stable, by allowing the birth terms, β_hi_, to consist of the deaths in all compartments. The rate at which hosts are bitten depends on the frequency at which tsetse take blood meals, and the relative preference for human and non-human animals. The probability of biting a human for N_v1_ is:

$$\theta_{h,1}=\frac{\sigma_{h}\left(N_{h1}+\left(1-\xi \right)N_{h2}\right)}{\sigma_{h}\left(N_{h1}+\left(1-\xi \right)N_{h2}\right)+\sigma_{a1}N_{a1}}$$

which can also be specified for the two human populations exposed to the vector population:

$$\theta_{h1,1}=\frac{\sigma_{h}N_{h1}}{\sigma_{h}\left(N_{h1}+\left(1-\xi \right)N_{h2}\right)+\sigma_{a1}N_{a1}}$$

$$\theta_{h2,1}=\frac{\sigma_{h}\left(1-\xi \right)N_{h2}}{\sigma_{h}\left(N_{h1}+\left(1-\xi \right)N_{h2}\right)+\sigma_{a1}N_{a1}}$$

and for non-human animals:

$$\theta_{a,1}=\frac{\sigma_{a1}N_{a1}}{\sigma_{h}\left(N_{h1}+\left(1-\xi \right)N_{h2}\right)+\sigma_{a1}N_{a1}}$$

where σ_i_ represents the relative preference for human and non-human host types. The probability of biting a human for N_v2_ is:

$$\theta_{h,2}=\frac{\sigma_{h}\xi N_{h2}}{\sigma_{h}\xi N_{h2}+\sigma_{a2}N_{a2}}$$

and of biting a non-human animal:

$$\theta_{a,2}=\frac{\sigma_{a2}N_{a2}}{\sigma_{h}\xi N_{h2}+\sigma_{a2}N_{a2}}$$

The dynamics of infections in animals are given by,

$$\frac{dS_{ai}}{dt}\;=\beta_{ai}+\;r_{4}R_{ai}\;-\;\frac{b\;f\;\theta_{a,i}\;I_{vi}\left(t\right)}{N_{ai}}\;S_{ai}\left(t\right)\;-\mu_{ai}S_{ai},$$

$$\frac{dI_{ai}}{dt}\;=\frac{b\;f\;\theta_{a,i}\;I_{vi}\left(t\right)}{N_{ai}}\;S_{ai}\left(t\right)\;-\;(\mu_{ai}+\eta )\;I_{ai},$$

$$\frac{dA_{ai}}{dt}=\;\eta \;I_{ai}-\;(\mu_{ai}+s_{ai})A_{ai},$$

$\frac{dR_{ai}}{dt}\;=\;s_{ai}A_{ai}-\;(\mu_{ai}+\;r_{4})R_{ai},$ where i is either 1 or 2. As for humans, the population sizes of animals are assumed to be stable, by allowing the birth terms, β_ai_, to consist of the deaths in all compartments.

The forces of infection on vectors are:

$$\Lambda_{v1}=\;cf\theta_{h1,1}\frac{A_{h1}}{N_{h1}}S_{v1}+\;cf\theta_{h2,1}\frac{A_{h2}}{N_{h2}}S_{v1}+c_{a1}f\theta_{a,1}\frac{A_{a1}}{N_{a1}}S_{v1}=\;f\;\frac{c_{a1}\sigma_{a1}A_{a1}+c\;\sigma_{h}(A_{h1}+\left(1-\xi \right)A_{h2})}{N_{a1}\sigma_{a1}+\sigma_{h}(N_{h1}+\left(1-\xi \right)N_{h2})}S_{v1}$$

$$\Lambda_{v2}=\;cf\theta_{h,2}\frac{A_{h2}}{N_{h2}}S_{v2}+c_{a2}f\theta_{a,2}\frac{A_{a2}}{N_{a2}}S_{v2}=\;f\;\frac{c_{a2}\sigma_{a2}A_{a2}+c\;\sigma_{h}\xi \;A_{h2}}{N_{a2}\sigma_{a2}+\sigma_{h}\xi \;N_{h2}}S_{v2}.$$

In the models where animals cannot get infected, *A*_*a1*_ = *A*_*a2*_ = 0, or equivalently, *c*_*a1*_ = *c*_*a2*_ = 0, in the equations above and tsetse flies can only get infected from humans although they can also bite animals. The ordinary differential equations describing changes in the vector compartments (with i indicating population 1 or 2) are:

$$\frac{dSvi}{dt}\;=\;\beta_{vi}\left(t\right)-\;\mu_{v}S_{vi}\left(t\right)\;-\Lambda_{vi}\left(t\right)$$

$$\frac{dEvi}{dt}\;=\Lambda_{vi}\left(t\right)-\left(\mu_{v}+v_{e}\right)E_{vi}$$

$$\frac{dIvi}{dt}\;=\;v_{e}E_{vi}-\mu_{v}I_{vi}$$

For ease of fitting and analysis we allow for the simplification that the birth terms β_vi_ consist of the death terms of all compartments, thereby ensuring stable tsetse populations. The impact of including tsetse population dynamics, density-dependence, migration and seasonality will be considered in a follow-up paper.

### Estimates of parameter sets

We obtained parameter sets using a Bayesian framework of importance resampling [43,44]. This entailed defining uniform ranges for parameter values; generating 50000 random samples of sets of parameter estimates drawn from the uniform priors (the same set of random samples was used for the six different scenarios—except for *c*_*a1*_ and *c*_*a2*_ which were set to 0 for the scenarios without animal reservoirs); running the model for 400 years (in order to avoid resampling simulation runs that move toward their equilibrium state at very slow rates) and obtaining a measure of the goodness of fit using a binomial likelihood function:

$$L_{i}=\prod_{j=1}^{14}\left(\begin{array}{c} N\\ x \end{array}\right)p^{x}{\left(1-p\right)}^{N-x}$$

where N is the human population size, *p* the target prevalence levels associated with high, medium or low transmission, and *x* is the simulated number of infected humans at *j* times 10 thousand days for the simulation run with parameter set, *i*; and randomly sampling 500 parameter sets from these 50000 proportional to their likelihood,

$$\Delta L_{i}=\frac{L_{i}}{\Sigma L}$$

which are samples from a distribution that forms an approximation of the posterior distribution. Based on these resampled parameter sets, questions of interest can be investigated such as the impact of vector control or screen and treat on prevalence over time.

### Basic reproduction number

The basic reproduction number, *R*_0_, is defined as the number of secondary infections that arise from a single infected case in a fully susceptible population. It provides insight into whether a pathogen can invade a population, and into which parameters of the disease system to target with control interventions [59]. We derive *R*_0_ using a next-generation matrix approach [60]. To do so, we separate the Jacobian matrix of the system into T, a matrix with the terms denoting infection events, and Σ, a matrix with the stage transitions (due to progression through the incubation period, death, etc.). A 16x6 matrix E of zeroes with a 1 in each column at the row corresponding to the infection events in T is also specified. The next-generation matrix K is then given by –E’T Σ^-1^E:

$$K\;=\;\left[\begin{array}{cccccc} 0 & 0 & 0 & 0 & k_{15} & 0\\ 0 & 0 & 0 & 0 & k_{25} & k_{26}\\ 0 & 0 & 0 & 0 & k_{35} & 0\\ 0 & 0 & 0 & 0 & 0 & k_{46}\\ k_{51} & k_{52} & k_{53} & 0 & 0 & 0\\ 0 & k_{62} & 0 & k_{64} & 0 & 0 \end{array}\right]$$

where the elements k_ij_ represents the average number of infections (of vectors or hosts of population i) arising over the infectious lifetime of one individual of host type j. The columns *j* correspond to A_h1_, A_h2_, A_a1_, A_a2_, I_v1_, and I_v2_, respectively. The basic reproduction number, *R*_0_, is equal to ρ(K)^2^, the spectral radius or dominant eigenvalue of the next generation matrix, squared to reflect the interest in transmission from host to vector to host.

## Supporting Information

S1 Table Rate parameter descriptions, values used and ranges for the model versions allowing for animal reservoirs. (DOCX)
