# Supplementary material for: Implications of Heterogeneous Biting Exposure and Animal Hosts on Trypanosomiasis brucei gambiense Transmission and Control
Source: PLoS Comput Biol. 2015 Oct 1;11(10):e1004514. doi: 10.1371/journal.pcbi.1004514 (PMC4591123; doi:10.1371/journal.pcbi.1004514)
Supplement: S1 Table — (DOCX) [file pcbi.1004514.s001.docx]

**Table S1: Rate parameter descriptions, values used and ranges for the model versions allowing for animal reservoirs**

| *Parameter* | *Description* | *Unit* | *Prior range* | *Median values by transmission intensity* | | |
| --- | --- | --- | --- | --- | --- | --- |
|  |  |  |  | High | Moderate | Low |
| μ_v_ | death rate of tsetse | Day^-1^ | [0.014-0.047] | 0.035 | 0.032 | 0.037 |
| f | inverse of duration of feeding cycle | - | [0.2-0.5] | 0.33 | 0.29 | 0.36 |
| σ_h_ | biting preference for humans | - | [0-1] | 0.14 | 0.23 | 0.63 |
| σ_a1_ | biting preference for animal type 1 | - | [0-1] | 0.68 | 0.46 | 0.24 |
| σ_a2_ | biting preference for animal type 2 | - | [0-1] | 0.46 | 0.81 | 0.04 |
| ξ | proportion of time spent in second region by commuters | - | [0-1] | 0.47 | 0.28 | 0.24 |
| b | proportion of infective bites leading to infection in humans and animals | - | [0-1] | 0.33 | 0.16 | 0.037 |
| c_h_ | proportion of bites on an infective human that lead to a mature infection in flies | - | [0.0001-0.0051] | 0.0034 | 0.003 | 0.0041 |
| c_a1_ | proportion of bites on an infective animal of type 1 that lead to a mature infection in flies | - | [0.0001-0.0051] | 0.0025 | 0.0033 | 0.0013 |
| c_a2_ | proportion of bites on an infective animal of type 2 that lead to a mature infection in flies | - | [0.0001-0.0051] | 0.0031 | 0.0021 | 0.0044 |
| v_e_ | 1/extrinsic incubation period | Day^-1^ | [0.025-0.0556] | 0.043 | 0.038 | 0.05 |
| η | rate at which hosts move from the incubating stage | Day^-1^ | [0.05-0.1] | 0.078 | 0.07 | 0.06 |
| s_1_ | rate of progression to stage II in humans | Day^-1^ | [0.0012-0.0028] | 0.0019 | 0.0021 | 0.0014 |
| s_ai_ | rate of progression to the immune class in animal hosts | Day^-1^ | [0.0012-0.0028] | 0.002/0.0019 | 0.002/0.0019 | 0.0026/0.0024 |
| μ_s1_ | disease-induced death rate / rate of leaving the recovered state for humans | Day^-1^ | [0.013-0.0029] | 0.002 | 0.0022 | 0.0026 |
| μ_h1_ | death rate of humans due to natural causes | Day^-1^ | [3.4-6.8e-05] | 4.63e-05 | 6.5038e-05 | 6.2691e-05 |
| μ_ai_ | death rate of animal host i | Day^-1^ | [0.000164-0.0027] | 0.0017/0.0015 | 0.0018/0.0011 | 0.0008/0.0025 |
| r | removal rate of infected humans due to treatment | Day^-1^ | [0.0006-0.0044] ^†^ | - | - | - |
| r3 | rate at which treated humans return to the susceptible class | Day^-1^ | [0.014-0.14] ^†^ | - | - | - |
| r4 | rate of loss of immunity in animal hosts | Day^-1^ | [0.002-0.0054] | 0.0034 | 0.0052 | 0.0025 |
| N_1_:N_2_ | ratio of humans in the low exposure environment to high exposure | - | [0-1] | 0.13 | 0.35 | 0.0004 |
| V:H_i_ | number of vectors per human in area i | - | [0-10] | 1.66/6.08 | 6.56/3.39 | 1.14/9.55 |
| A:H_i_ | density of animals relative to humans | - | [0-2] | 1.14/0.79 | 1.55/1.04 | 0.47/0.79 |

^†^Values were zero (and not fit to prevalence levels) unless the interventions of screening and treatment of humans was simulated
